# Implementation strategies to support pre-exposure prophylaxis for HIV prevention care for cisgender women in federally-funded family planning clinics in Atlanta, Georgia

**DOI:** 10.3389/frph.2025.1529067

**Published:** 2025-09-02

**Authors:** K. M. Anderson, L. V. Bonadonna, E. J. Cortes, D. L. Er, C. K. Ellison, P. Williams, S. S. Sullivan, M. W. Brooks, A. N. Sheth, J. M. Sales

**Affiliations:** ^1^ Department of Behavioral, Social, and Health Education Sciences, Rollins School of Public Health, Emory University, Atlanta, GA, United States; ^2^ Department of Medicine, School of Medicine, Emory University, Atlanta, GA, United States; ^3^ The Family Health Centers of Georgia, Inc., Georgia Family Planning System, Atlanta, GA, United States

**Keywords:** HIV prevention, PrEP, family planning, Title X, cisgender women

## Abstract

**Introduction:**

Title X-funded family planning clinics stand to play a significant role in Ending the HIV Epidemic (EHE), as a unique access point for cisgender women in the U.S. who account for approximately 1 in 5 new HIV infections. Biomedical HIV prevention, known as PrEP, is effective for cisgender women, yet uptake remains low and rates of new infections among women have remained steady while other key populations have experienced declines. Further, significant racial disparities persist in PrEP uptake and HIV acquisition, with Black women accounting for almost 60% of new cases. Low risk perception, lack of knowledge, and insufficient access to biomedical HIV prevention in sexual health service settings contribute to this. Despite receiving federal funds to provide free and low-cost family planning and preventative sexual health services, Title X clinics do not routinely provide PrEP as part of their services; this excludes the millions of U.S. women who engage in sexual behaviors aligned with HIV risk from access to effective prevention when utilizing Title X clinics for care.

**Methods:**

In the course of developing a systems-level bundle of tailored implementation strategies to support PrEP care delivery in Title X-funded family planning clinics, we conducted a series of baseline focus groups with staff and providers at Title X family planning clinics located in 4 EHE priority jurisdictions that comprise metro Atlanta. The analysis of these focus groups aimed to elucidate important considerations and needs to inform implementation strategy development and strengthen PrEP care delivery in these safety-net clinics.

**Results:**

This article describes the findings from these focus groups and recommendations and next steps for scaling PrEP in Title X family planning clinics in the Southern U.S. to improve PrEP reach among cisgender women.

## 1 Introduction

Family planning clinics stand to play a significant role in Ending the HIV Epidemic (EHE) for cisgender women in the United States (U.S.) (1–4), a population accounting for approximately 1 in 5 new HIV infections yet systematically under accessed for HIV prevention (5). Rates of HIV infection for cisgender women have remained stagnant in recent years, even as rates among other key populations have fallen (6); Black women alone account for 54% of new diagnoses among U.S. women (6).

These new infections are preventable with biomedical HIV prevention methods, including three modalities approved for women: daily oral PrEP in the form of pill, bimonthly injectable PrEP administered by a health care providers, and twice-yearly injectable PrEP, administered by healthcare providers (7–10). Yet, uptake by women and minoritized individuals is lacking (5, 11, 12). Of those who could benefit from PrEP, only 13% of Black individuals and 15% of women assigned female at birth had been prescribed PrEP, as of 2022 (12). Behavioral HIV prevention (e.g., internal and external condom use) can prevent HIV transmission through heterosexual contact, which accounts for the majority of new cases among cisgender women (5). However, behavioral prevention has variable levels of efficacy (7, 13–15), and cannot provide protection from other pathways of transmission, such as injection drug use (16). Further, less than 20% of women endorsed consistent condom use in the 2011 to 2014 National Survey of Family Growth (NSFG) (17), while men who have sex with women reported decreased condom use between 2002 and 2017 (18). Lack of knowledge and low HIV risk perception limit uptake of HIV prevention methods among women, including those at risk through heterosexual relationships (19–22); improving knowledge of HIV prevention is key to increased uptake of both behavioral and biomedical prevention options (23, 24). Universal preventative HIV counseling is recommended for cisgender women (2, 25), is effective (26, 27), and is acceptable in a clinical setting (3, 28, 29).

Clinics funded under Title X receive federal funds to provide free and low-cost family planning and preventative reproductive health services, and annually serve over 100,000 women in Georgia alone (30), —the U.S. state with the highest rate of new HIV infections, over half of which occur in the capitol of Atlanta (11). Eighty percent of women attending Georgia Title X Clinics are estimated to be at risk for unintended pregnancy, indicating sexual contact without a barrier method which could lead to HIV transmission (30). Due to their high potential reach for HIV prevention among cisgender women, family planning settings have faced numerous calls for integration of women-centered HIV prevention interventions (3, 4) and PrEP provision (1, 2) and are documented as an acceptable venue for HIV prevention activities (3, 28, 29). Further, the body overseeing the Title X program- the Office of Population Affairs- recommends use of evidence-based preventative screening and counseling, as well as service provision that includes HIV/AIDS services (31). Despite this, HIV prevention programs- including programs for assessment of eligibility, prescribing, and follow-up care associated with PrEP- are underdeveloped in Title X programs, particularly in the Southeastern U.S. (32). This represents a significant missed opportunity in efforts to End the HIV Epidemic (33).

In the course of developing a systems-level bundle of tailored implementation strategies to support PrEP care delivery in Title X-funded family planning clinics in Georgia, we conducted a series of baseline focus groups with staff and providers at Title X family planning clinics in the metro Atlanta area. The analysis of these focus groups aimed to elucidate important considerations and needs to inform implementation strategy development and strengthen PrEP care delivery in these safety-net clinics, expanding upon the limited available research on PrEP implementation in Title X clinics.

## 2 Materials and methods

### 2.1 Setting

The epicenter of the U.S. HIV epidemic is the U.S. South. In the city of Atlanta alone, the rate of new HIV infections (44 per 100,000) is higher than the rate of new HIV diagnoses in Georgia (28 per 100,000), the U.S. Southern region (19 per 100,000), and the U.S. as a whole (14 per 100,000) (11). The four counties comprising the Atlanta metro area (Cobb, DeKalb, Fulton, and Gwinnett) are each identified in the *EHE* Initiative as priority counties (33), indicating high rates of transmission and particularly need for prevention efforts. Within these four counties there are 31 Title X Clinics, overseen by the Georgia Family Planning System (GFPS), representing approximately 20% of Title X Clinics in Georgia (30).

### 2.2 Procedures

This work was conducted as part of a larger study to develop, implement, and evaluate tailored implementation strategies to support PrEP delivery at metro Atlanta Title X clinics. Between January of 2022 and November of 2023, 47 members of clinic leadership and staff from eleven clinic groups representing 22 of 31 Title X Family Planning clinics (71%) in the metro Atlanta area participated in baseline (pre-implementation) focus groups facilitated by graduate-level researchers with training in qualitative methods. The aim of these focus groups was to gather information regarding current HIV testing practices, PrEP care processes at their clinics, and to gauge acceptability and interest in PrEP capacity-building activities tailored towards their clinical needs. While most Title X clients are female, Title X clinics provide sexual and reproductive health care to all individuals, therefore capacity strengthening efforts encompass services to all individuals, not just cisgender women, and focus group content reflects this. Eligible clinic groups (e.g., received Title X funds and had clinical sites located in Metro Atlanta) were invited to a pre-implementation focus group with the study team. Focus group participants were presented with a menu of trainings, technical assistance (TA), and patient- and provider-facing resources (Table 1) developed by the study research team and designed to support implementation of PrEP care in Title X clinics. Additionally, facilitators used a structured focus group guide with questions structured by deductive implementation-focused domains (Table 2) to elicit responses on targeted key topics, as is common practice in rapid qualitative methods (34). Domains were identified in line with the updated Consolidated Framework for Implementation Research (CFIR 2.0) (35) including “Existing PrEP Landscape”- encompassing CFIR 2.0 Implementation Process Domain constructs such as *assessing needs, assessing context, planning, engaging, doing,* and *adapting-* “Capacity for Change- Needed Resources and Trainings”- corresponding to Inner Setting Domain constructs such as *available resources, compatibility,* and *access to knowledge and information*- and “Preferences for Resources and Training,” aligned with Innovation Domain constructs of *innovation adaptability, innovation complexity,* and *innovation design.* Based on the content of the focus groups, clinic groups could identify the number of clinic leadership and staff they felt should be invited to participate in a focus group to provide comprehensive information for their clinic group. Thus, clinics determined the number of individuals that would be best suited to speak to their sites' HIV testing and prevention services. All focus group participants worked at Title X Family Planning clinics in the metro Atlanta area and were able to provide verbal consent to participate. Focus groups (*N* = 12) were conducted in-person (*n* = 1) or via Zoom Videoconferencing (*n* = 11) and lasted an average of 38 min. All participants completed informed, verbal consent prior to participation in accordance with an IRB determination of “minimal risk.” Verbal consent was documented by the staff member conducting the informed consent. A note-taker was present at each focus group, and focus group discussions were recorded, then sent to an external transcription service. Staff members checked transcriptions against the original recordings and removed identifying information. Focus group participants each received a $50 gift card for their participation. All procedures were approved by the Institutional Review Board at Emory University (STUDY00002950).

**Table 1:** Menu of PrEP-related trainings, technical assistance, and resources offered to title X clinics.

Training title	Training description
Overview of HIV Testing and PrEP	General overview of PrEP (what it is, how it works, potential benefits); tips for conversations about PrEP; overview of HIV testing (universal, opt-out, routine) geared towards non-clinical staff
PrEP Care for Clinicians	Steps for PrEP implementation including interpreting HIV tests and labs, medication dosing and prescribing PrEP, monitoring & follow-up, and women's considerations
PrEP Insurance Navigation	How to navigate Georgia insurances/medication assistance programs for PrEP
PrEP: What's on the Horizon	New products available and those in development (ex., long-acting PrEP)
Alternative PrEP Delivery	Overview of PrEP care models (i.e., same-day &telehealth PrEP)
Reporting PrEP Metrics	How to report PrEP metrics for the Title X Family Planning Report
Taking a Sexual History	Improving skills in taking a detailed, non-judgmental sexual history
STIs (2021 Guidelines)	Review CDC 2021 STI diagnosis and management guidelines
Technical Assistance	
Follow-in support for any PrEP trainings	Individualized, tailored follow-up and hands on support specific to any of the trainings offered
PrEP implementation planning consult visit	To facilitate the development of a plan for PrEP implementation in their clinic
PrEP and EHR consultation	To provide technical assistance on how to incorporate PrEP care steps into the EHR and/or utilize the EHR to track PrEP metrics
Consultation on alternative PrEP delivery	To assist clinics who want to incorporate novel PrEP delivery models (ex.: same day PrEP & telehealth PrEP)
PrEP Resource Materials	
Patient-facing PrEP materials	Flyers, handouts, posters including with specific content for women (available in multiple languages)
Provider-facing materials	Reference sheets on HIV testing, PrEP counseling, and/or PrEP steps, and sexual health assessment
Sexual health assessments	Standardized sexual health assessments that can help identify patients who may most benefit from PrEP, inclusive of unique considerations for women
Opt-out HIV testing guidance	Template example of opt-out of HIV testing consents

**Table 2:** Deductive domains and key points relating to PrEP implementation in title X clinics in Atlanta, GA, USA.

Domain	Key points
Existing PrEP Landscape	• Providers had few patients on PrEP, but recognized the capacity and desire to increase PrEP availability & uptake • Providers perceived patients to be uninterested or lacking risk perception • Identification of new PrEP patients was attributed to provider discomfort and (lack of) current protocols • PrEP retention within 90-day was limited, despite significant efforts • Cost was considered a barrier to PrEP uptake and retention, both for medication and appointments/testing
Capacity for Change—Needed Trainings and Resources	• PrEP capacity could be improved by increased educational efforts in clinics • Trainings on how to take a sexual history and alternative PrEP delivery were cited as most helpful • Providers recommended offering multiple trainings on specific topics within PrEP provision, as well as resources • Participants also understood that there would be different trainings needs for different staff members and providers • Resources for providers (e.g., reference sheets) and for patients (e.g., flyers) would complement trainings to bolster capacity
Preferences for Resources and Training	• For non-training informational resources, participants recommend they be surface-level, compact, and easily accessible • Training should be 30 min or less and occur in group settings • In-person training was preferred, but the utility of offering a virtual option was noted • Resources should be available in both virtual and physical formats

### 2.3 Analysis

Graduate-level researchers with training in qualitative methods employed a multi-stage rapid analytic method. Rapid analysis of the data was selected to facilitate time-sensitive identification of needs and considerations for implementing trainings, as is appropriate for qualitative work informing interventions (36, 37). A modified RADar technique (38) was used for this analysis. First, a rapid analysis matrix was created with focus group IDs along the vertical axis and focus groups questions along the horizontal axis, grouped by five implementation-focused domains: community population, existing HIV testing landscape, capacity for change relating to HIV testing, existing PrEP provision landscape, capacity for change relating to PrEP provision, implementation logistics, and capacity for evaluation. Though all five domains were covered, the focus of this analysis is specific to existing PrEP provision landscape. resource-related capacity for change related to PrEP provision, and qualities of resources to facilitate implementation. One researcher populated the matrix using the response to each question from each focus group; the populated matrix was reviewed by a second researcher for agreement. Then, the second researcher reviewed responses across each question (i.e., each column), combining questions into their larger domains when appropriate, while removing extra language from responses that did not provide context or facilitate increased understanding. This revised document was reviewed by the first researcher for agreement and consistency of meaning from the original transcripts. Finally, the second researcher developed a narrative around the retained quotes, reporting key points and identifying demonstrative quotes. In line with implementation-focused rapid analysis, we considered deductive domains to have key points that are informative for implementation support (34), rather than themes and sub-themes. The narrative was iteratively reviewed by both researchers involved in the rapid analysis, and the larger study team, and revised for clarity. Results are grouped by the deductive domains as structured in the focus group guide; Table 2 presents the deductive domains with key points within each. Focus group number is identified in parentheses following each quote, with respondent profession when available.

## 3 Results

### 3.1 Participants

Focus groups had between 1 (*n* = 1) and 20 participants (*n* = 1), with an average of approximately four participants. Eligible participants worked at Title X-funded Family Planning clinics in the state of Georgia and were able to provide verbal consent to participate. Participants included anyone with the potential ability to prescribe PrEP and counsel or screen for HIV prevention services, including health educators/patient navigators/program coordinators (26%), physicians (21%), mid-level providers (13%), nurses (13%), medical assistants (15%), and clinic administrators (12%).

### 3.2 Existing PrEP landscape

Providers generally endorsed having few, if any, patients on PrEP, but recognized the capacity for increasing PrEP uptake as well as a desire to increase PrEP offerings within their clinics. One participant mentioned, “… we haven't seen a whole lot of upticks … with PrEP and really talking with patients about it. … I think there are definitely opportunities in our clinics to do that a little bit more,” (FGD9, Administrator) while another asserted, “We're always looking to get more patients in, [but] we don't have a lot of patients on PrEP right now. I don't think I have any cisgender women on PrEP. Actually, I don't think I have any women on PrEP right now” (FGD7, Clinician).

Providers shared that they perceived their patients to be uninterested, or that patients didn't perceive themselves to be at risk of HIV. “Maybe it might be our population, but they may not be as interested” (FGD2, Clinician) one shared, while another reflected on how the framing of HIV risk that has promoted this lack of engagement:

In some of our meetings, a lot of heterosexual men don't feel PrEP is for them. So, one of the things that I find is that a lot of the advertising for PrEP has been mostly showing MSM, and partially because it's a higher rate. Unfortunately, that's putting a stigma out there that heterosexual men don't need PrEP. … some patients they all say, “Oh, I don’t need that. I’m not gay.” (FGD4, HIV Services Coordinator)

Those patients that *did* come in specifically requested PrEP, while participants said that those introduced through their clinic were generally not interested day-of; it was unclear if a previous visit may have prompted subsequent PrEP requests. One provider commented “…if I have someone coming in, they're not coming in for PrEP but they have a history of multiple STDs… then it will be something that I mentioned or that I introduced them to, so that they can say oh, that does fit me or that doesn't. I haven't had anyone say after I introduced them, yes, I would like that. So far, the PrEP patients that are coming in are PrEP patients coming in” (FGD7, Clinician).

Others considered the possibility that providers and current protocols, or lack thereof, were contributing to the lack of newly identified PrEP-eligible patients. One participant discussed that “we don't really see a lot of them [potential PrEP patients] through our [non-protocolized sexual history] screening” (FGD2, Clinician), while another commented, “the testing, that's the easy part, we can get everyone tested… But now, interpreting those results, following up with patients, and starting that conversation with patients, or identifying the patients that are at high risk [are more difficult]- how to be able to take that information or screen the patients, so that we can identify the patients that will benefit from that” (FGD1, Clinician). Discomfort starting the conversation, or provider comfort more generally, was brought up by several participants as a potential barrier; one provider questioned, “Is it that, you know, from a clinical perspective, perhaps, there is not that conversation, or they're just not comfortable? … it has just been really challenging to understand why that has not been prescribed, and that's why I think that's important to have the resource and the education” (FGD1, Clinician). Another participant endorsed her own limitations in this regard, sharing, “And like I said, [some of] our providers… aren't fluid in asking these types of questions. … I've been out [of school] for quite some time as far as training and the other things” (FGD5, Clinician).

For patients who were interested in PrEP, providers noted significant barriers to retention, particularly at the 90-day/3-month follow-up, despite sometimes significant efforts to facilitate PrEP retention through having multiple pathways to obtain medication and attend clinic visits. A participant shared,

I've tried to kind of do some research to see what's the main purpose of the patient not coming back, because what I do is when they prescribe PrEP I call them after a week and check up on them to see how everything is going. And then I'll call them a month later, and then right at that 90-day. I haven't—we haven't gotten to the point of any patient being on the 90-day just yet through me. However, with the navigation I felt it could help them to be in compliance with what we're looking for and having them stay on PrEP or making sure that as they leave they can get it. So, we've set it up with the pharmacy to where they can come in or we can mail it, the PrEP out to them if they can't come and get it. And as far as coming here for the visit we have—right now we have a grant for Uber rides, so we'll provide Uber rides. (FGD4)

Cost was cited as one potentially significant barrier to PrEP uptake and retention, both for PrEP itself and the additional appointments and testing required to remain on PrEP. One participant commented that regarding PrEP medication itself, “they've… pulled back on prescribing Truvada and now they give the generic. Unfortunately, the programs don't assist with generic …. Now, insurance covers that, but again, the uninsured…” (FGD4, HIV Services Coordinator). Another discussed the financial burden associated with follow-up visits, saying,

…the other thing that I've run into is for patients that are on the sliding fee scale, they might have an issue with paying that bottom dollar amount that we normally charge for them to come. … I'm looking for a grant or some assistance for us to be able to pay for that visit when they come back, at least for the first nine months to a year, to pay for those every-three-month visits. (FGD4)

### 3.3 Capacity for change—needed trainings and resources

Capacity to provide PrEP could be improved by addressing some of these barriers, such as beginning conversations about PrEP, cost and insurance navigation, and adherence or retention in PrEP, through increased educational efforts in clinics. When asked about such opportunities, respondents cited interest in trainings on PrEP- “Provider training is obviously something we need” (FGD2)- and discussed the limited availability of trainings they have run up against. One participant said their clinic had “been searching hard. We've been going all the way to California—online, not in person—to identify resources and so forth that are readily available [PrEP and HIV resources]. But it's been hard…” (FGD2, Medical Director).

Interview participants were supplied with a list of potential trainings that could be provided to them (Table 1). Specific trainings were frequently mentioned as being of particular interest or applicability, including alternative PrEP delivery methods and how to take a sexual history. Taking a sexual history was noted as potentially beneficial by the largest number of participants, with frequent mention of the potential utility of such a training for “support staff,” and “medical assistants,” in particular. Important points of this training could include “how to navigate through that history without any judgment…” (FGD4, Clinician), “[guidance] to understand if, you know, that individual, that patient, would benefit from the services of PrEP” (FGD1, Family Planning Lead), allow staff to feel “equipped and ready to handle this patient population” (FGD7, Medical Director). The next most frequently cited was alternative PrEP delivery; however, what participants included in their definition thereof did vary. Examples included “using telehealth appointments to incorporate—to get PrEP out to patients or PrEP education (FGD2, Health Programs Coordinator)” “[providing] that prescription… the same day,” (FGD1, Family Planning Lead) and “the injectable PrEP option” (FGD11).

Additionally, participants noted a few other trainings of particular interest; two identified reporting on PrEP as potentially beneficial, with one saying, “I could sit through the reporting PrEP metrics and …probably could transfer over to helping me, which is the overall center” (FGD4, HIV Services Coordinator). Conflicting information was provided about the utility of insurance training- two participants had opposite views, with one stating it wasn't a priority as existing support was already available. Several other trainings, including how to have conversations about PrEP, PrEP for clinicians, and integration of PrEP into existing workflow, such as the electronic medical record, were mentioned once by different participants. Beyond these training of interest, several participants cited that multiple trainings on specific topics within PrEP provision, as well as resources, would be helpful:

This is kind of, is exactly what we are looking at, to have a systematic approach to the whole process. The trainings that you have listed here is exactly what we are looking at, what the clinicians and the providers are looking for, information on how to implement this, the monitoring, the follow-ups. (FGD3, Clinician)

Another shared that “they would all be helpful. I know I was specifically interested in, the PrEP care for clinicians, the insurance, navigation, alternative delivery, and the new products that are available,” (FGD7, Clinician) while a third noted they may not need some specific training, “but also, who turns down resources?” (FGD7, Medical Director). Participants also understood that there would be different trainings needs for different staff members and providers:

So, we understand there's going to be tiers. There will be a very dedicated HIV team training—specialized, technical—but we also want the entire health center staff to become more familiar, to be more aware, and to be more expert level. They'd better know better than the patients what can happen. So, we want to definitely provide training to raise the level of understanding and the knowledge of the entire staff for our health center. (FGD2, Medical Director)

Complementary to trainings, resources for providers (e.g., reference sheets) and for patients (e.g., flyers) were discussed as advantageous for the implementation of PrEP. One respondent shared they liked informational materials for patients “because oftentimes you can give that to them on the initial visit. They're kind of slow about the response. You can give them that to take home and look at, and then it would be on the next visit” (FGD4, Clinician). Additionally, “for new providers or even providers who are hesitant about ordering PrEP and things like that because they just refer them to the PrEP clinic, it might be a good idea to have the reference sheets for them as well” (FGD9, Clinic Administrator).

Participants were asked what else was important to train on or provide resources regarding. Two participants mentioned the importance of information for adolescents and young adults:

I've—most of what I've come across has been things that tailor to adults. And because the data is from 13 to 65, a 13-to-18/19-year-old doesn't see things how an adult would, 30—whether it's 18 you're an adult. So, I don't really have, or I haven't found much that really talks to a younger person from being sexually responsible to creative ways to protect yourself. I mean, that's one of the things that I'm now researching as well, trying to find a way to communicate to the younger generation. (FGD4, HIV Services Coordinator)

Several others cited the need for materials that are more accessible, meaning available in different languages, requiring low literacy levels, and appropriate for different cultures. Participants commented, “…having that in English and Spanish, that's one of the things …that's big, you know, having it in Spanish and being able to give that information as well,” (FGD9) and “… more so than African, [the clinic sees] West Africa refugees that we have in our population. [Do they] have any translated materials in languages like Somali…?” (FGD10) One participant pointed out, “The other question is, a good number of our population are also illiterate. So do you have any low literacy type education materials surrounding this?” (FGD10, Nurse Administrator).

Other things participants said were needed included refresher courses, information on post-exposure prophylaxis (PEP), and ways to increase understanding of HIV risk while decreasing stigma. Regarding the last point, one practitioner shared, “There's a feeling that [PrEP is] like a gay medication. And I've had discussions with, again, with other groups to try to—to see if they feel that same way. And they do. And we're—and now we're trying to discuss how do you close the gap and get everyone the same understanding that it's needed if you have multiple partners or are sharing needles or whatever you're doing. So, I don't know if there could something to kind of give—to promote that it's for both. But that's another challenge.” (FGD4, HIV Services Coordinator).

### 3.4 Preferences for resources and training

Beyond the types and content of trainings and resources needed to scaffold successful PrEP implementation, participants were asked about the mediums and qualities of trainings that would fit into their clinics most easily. For non-training informational resources, participants recommend they be surface-level and compact, as well as easily accessible within clinic spaces, such as waiting rooms or informational bulletin boards. One participant shared,

I don't think we need a more in-depth. I think we need a more general, just general information to have around the clinic like in the waiting area, in the exam room. I think from a provider standpoint we understand, but we have new employees. We have patients. While they wait they read information. So it sparks a conversation. (FGD8, Clinician)

Training, specifically, was preferred to be 30 min or less, available in-person or remotely via videoconference, and to occur when entire clinical groups were together, usually in pre-determined team meeting times. One participant reflected,

I do like having training and just getting people to understand more about PrEP. …. I do like the fact that it's more truncated. You have it like 30 min vs., you know—because it is during lunchtime, so if there's a way to do that. Also just making sure that their time is useful, and you can do it—I guess you may come onsite, but then also, you know, having the ability to do it during WebEx or Zoom or something like that too. (FGD9, Administrator)

Time efficiency was highly valued, given provider scheduling restraints. Participants shared that shortened trainings helped to address one of the major barriers to hosting learning activities- limited time availability.

One of the challenges with… doing training with the providers is they do meet weekly, but obviously their calendars get pretty full and they meet for one hour. So to have trainings with them they do need to be shorter. So I saw that you guys had [proposed] where the clinician one was offered through 30 min sessions or one one-hour session. So that was really good for us. (FGD10, Family Planning Lead)

When possible, offering trainings to all offices within a clinic system was cited as potentially beneficial, particularly as there were often pre-specified times available for this. As one practitioner stated, “so normally, every last Friday of the month, we close early for training, and all the staff come to the one location in [town]. So …we can all be part of the training” (FGD1, Administrator). Another said, “We do—the first and third Wednesdays are our training days in the afternoons, so everyone is accustomed to coming to one location anyway” (FGD5, Medical Director). Regarding the format, in-person was generally preferred, but the utility of offering a virtual option was repeatedly noted- “So in-person or virtual is fine. I still give my providers and staff an option to do virtual as well. So we might be here in person, but I still have a Zoom link or, you know, a teams link attached for those who cannot make it in person” (FGD9, Administrator); “We're still utilizing technology for trainings. So on this we prefer hands on one-on-one training that's still available at each site, but most of our training is done via Microsoft Teams and we have everyone meet at that point and conduct trainings” (FCD10, Administrator).

Training materials and resources were generally wanted in both virtual and physical formats, though a few mentioned online training systems where materials could be hosted, or that some individuals may have a personal preference for virtual materials. One provider said, “having electronic versions of some of these provider-facing materials would just be good to have in your back pocket and then actual printed versions for the participants or the patients in the clinic in the lobbies would be really helpful,” (FGD9, Clinician) while another hedged, “I know [other respondent] said something as far as like a handout or kind of a chart that we have, a handheld chart. So I know some people are more tech savvy, they don't necessarily like to have paper, I think” (FGD5, Clinician).

## 4 Discussion

Cisgender women are largely under-equipped with HIV prevention strategies. To End the HIV Epidemic, we must work on expanding access to PrEP for all individuals at risk of acquiring HIV, meeting them where they are already seeking care. In this study, we explored barriers to PrEP implementation in Title X family planning clinics, cornerstone caretakers for women in the U.S., and assessed need for and interest in various capacity-building implementation strategies (e.g., educational trainings, technical assistance, and resources) to increase capacity to provide PrEP. Various barriers such as difficulty initiating sexual health conversations, insurance navigation, and follow-up visit costs were described in the existing Title X clinic landscape. Many participants expressed a need for time-effective PrEP screening and counseling procedures and training covering sexual history taking, destigmatizing PrEP, and medication access. Further, these clinics serve more than cisgender women for sexual health needs, thus the importance of content also inclusive of diverse populations, including men. Population-tailored information, such as non-English, youth-focused, and low literacy responsive materials were highly desired by clinic staff.

Our results reflect other studies on barriers to PrEP implementation for women in the United States. A systematic review found that knowledge of PrEP was low among women and medical providers, although willingness for patients to try it and providers to incorporate into medical practice was high (39). Gaps in provider-initiated PrEP counseling have been specifically reported in family planning clinics (40, 41), highlighting the need to focus on increasing education for staff and providers. Our findings indicate that family planning clinic staff would like continuing education on PrEP and are very receptive to collaboration with academic partners for these trainings. It is notable that desired trainings go beyond eligibility for PrEP, expanding to how to have conversations about sexual health, stigma, and other topics.

Several studies have designed interventions to increase PrEP uptake among women, including peer-navigators to increasing linkage to PrEP (42); web-based educational videos for patients (43); and, use of a dedicated a preexposure prophylaxis nurse in an urban obstetrics and gynecology clinic (44). Although these studies showed increased awareness and comfortability talking about PrEP, none showed statistically increased number of PrEP prescriptions. Systems-levels interventions hold promise for larger reach than individual-level interventions, while also removing the onus of information seeking and PrEP engagement from women. There is also preliminary support for the effectiveness of such interventions- one study with medical providers significantly increased PrEP prescriptions in one sexual health clinic in Washington D.C. by providing educational trainings to medical providers, electronic medical record prompts, and generalized informational videos to patients (45). Work expanding PrEP education to multiple clinics in high priority geographic areas, especially in the Southern U.S., are now needed.

To fill this gap, based on our prior research with Title X clinics in the Southern U.S. (46–48). coupled with the results of this study, we were able to refine our capacity-building implementation strategies, which ultimately consisted of a bundle of trainings (HIV, PrEP, and sexual health topics), patient- and provider-facing resources (in English and Spanish), and tailor (e.g., language, training delivery mode, training length and depth) them to the needs of this diverse network of clinics across Metro Atlanta. Further, based on this study's findings, the Title X clinical sites can then request the specific trainings, technical assistance and PrEP resources that meet their unique capacity and resource-related needs.

There are several limitations to our study. Only Title X staff from clinics located in the four EHE jurisdictions of metro-Atlanta were invited to participate, so this work may not be generalizable to more rural clinical sites in Georgia or other areas of the U.S. We also had variability across sites in the number of participants per focus group which may have resulted in missing perspectives from some clinics. Staff members who analyzed the data are academic researchers, not clinicians, therefore there may be interpretations that depart from a practitioner perspective. Despite these limitations, the majority (71%) of Title X clinics in metro Atlanta participated in these focus group discussions, so our sample does represent most of the Title X family planning network in this area.

Partnership between academic institutions and Title X family planning clinics is acceptable and has the potential to sustainably fill gaps in PrEP education needs for safety net clinics. The results of this study were used to prepare a systems-levels intervention with the primary aim of increasing PrEP uptake in four priority districts of metro-Atlanta. This intervention is currently ongoing, and results will be reported separately.

## Data Availability

The raw data supporting the conclusions of this article will be made available by the authors, without undue reservation.
